# Comparison of perioperative outcomes of single-port laparoscopy, three-port laparoscopy and conventional laparotomy in removing giant ovarian cysts larger than 15 cm

**DOI:** 10.1186/s12893-021-01205-3

**Published:** 2021-04-21

**Authors:** Xiaoying Wang, Yan Li

**Affiliations:** Department of Obstetrics and Gynecology, Shengjing Hospital of China Medical University, No.36 Sanhao Street, Shenyang, China

**Keywords:** Giant ovarian cysts, Single-port laparoscopy, Three-port laparoscopy, Laparotomy

## Abstract

**Background:**

Although conventional laparoscopy has gradually accepted as a surgical treatment for ovarian cancer, reducing the port numbers of laparoscopy still has great challenge for larger ovarian tumors. Thus, this study aims to explore the surgical outcomes of single-port laparoscopy for removing giant ovarian cysts (≥ 15 cm) and compare with laparotomy and three-port laparoscopy.

**Methods:**

This study enrolled 95 patients with giant ovarian cysts (> 15 cm) who underwent single-port laparoscopy, three-port laparoscopy or laparotomy. Their medical records, perioperative surgical outcomes, and postoperative pain score and complications were analyzed and compared retrospectively.

**Results:**

Single-port laparoscopy showed better perioperative outcomes and less postoperative pain than three-port laparoscopy and laparotomy. The time between post-surgery and getting out of bed in single-port laparoscopy was significant shorter than that in the laparotomy and three-port laparoscopy (17.53 ± 7.26 vs 29.40 ± 9.57 vs 24.56 ± 7.76, *P* < 0.01). The length of hospital stay in single-port laparoscopy was significantly shorter than that in other two groups (4.06 ± 0.5 vs 5.46 ± 1.63 vs 4.81 ± 0.83, *P* < 0.001). In addition, single-port laparoscopy had the lowest postoperative pain scores than in the laparotomy and three-port laparoscopy. There were no significant differences of total hospital cost, postoperative complications and time until gas passing among the three surgical groups. Importantly, in the removal of giant ovarian cysts, the proportion of cyst rupture in single-port laparoscopy was far lower than that in three-port laparoscopy (3.0 vs 22.2%).

**Conclusions:**

For giant ovarian cysts, single-port laparoscopy is still a safe and efficient technique with the advantages of short operation time, less estimated blood loss, short hospital stay, lower spillage rate, and less postoperative pain.

## Background

As laparoscopic surgery has gradually been accepted as a method of treating cancer, reducing the number of ports has become a trend to make the operation more minimally invasive and better cosmetic effects [1–3]. Even though ovarian tumors or cysts may grow very large, laparoscopy has been proven to be feasible for ovarian cyst larger than 10 cm [4]. It was reported that during laparoscopic surgery, patients with ovarian tumor large than 10 cm have more estimated blood loss, longer operation time and longer hospital days than patients with tumors < 10 cm [5]. With the development of three-port laparoscopy, it has displayed the same or even better short-term and pathological outcomes than five-port laparoscopy [3]. However, reducing the port number to single-port laparoscopy is a challenging and highly demanding technique. Recently, with the technique improvement, single-port laparoscopy has been successfully used to remove smaller ovarian cyst/tumors [6–8], and its operation time, estimated blood loss, would infection and postoperative pain are similar to that of laparotomy [9].

Although the aforementioned studies have shown that single-port laparoscopy can be successfully used to remove ovarian tumors, single-port laparoscopy for giant ovarian cysts may encounter some operational problems. When laparoscopic instruments are inserted in parallel through the single hole, due to the limited range of motion of the instruments, frequent collisions may occur, which may increase the dissection difficulties, operation time and cyst rupture. Especially when the ovarian cyst in the abdominal cavity is very large, the remaining small space further limits the flexibility of surgical instruments. Currently, there are few reports comparing the surgical outcomes of using single-port laparoscopy, three-port laparoscopy or conventional laparotomy to remove giant ovarian cysts, which is defined as the diameter > 15 cm. Thus, the aim of this study was to compare the perioperative outcomes and postoperative complication of patients with giant ovarian cysts who received single-port laparoscopy, three-port laparoscopy or laparotomy. Through this study, we can understand whether single-port laparoscopy still retains its advantages over three-port laparoscopy and laparotomy in removing giant ovarian cysts.

## Methods

### Study design and participants

This retrospective comparative study was approved by the Institutional Research Review Board of ShengJing Hospital of China Medical University, China. From January 2017 to December 2018, a total of 95 patients were diagnosed with giant ovarian cysts (> 15 cm) and received single-port laparoscopy, three-port laparoscopy or laparotomy. Patients were categorized according to the types of surgery received, including 33 cases of single-port laparoscopy, 35 cases of laparotomy, and 27 cases of three-port laparotomy. Medical records of these patients including age, body mass index (BMI), cyst diameter, previous abdominal surgery, CA-125 levels, ASA physical status classification, and tumor pathology were collected. In addition, perioperative and postoperative values of the patients were recorded. Operation time was defined as the time interval between umbilical incision and the completion of skin closure. Estimated blood loss was defined as the amount difference between irrigation and suction before and after surgery plus the difference of the gauze weight. Hemoglobin change was defined as the difference between the amount of hemoglobin on day 1 after surgery and day 7 before surgery. Length of hospital stay and total hospital cost were defined as the number of days and total cost from surgery to discharge, respectively. Time until gas passing was defined as the time interval from the post-surgery to the time when patients to have gas passing. The time until leaving bed was defined as the time interval between post-surgery and getting out of bed for activity.

### Surgical technique

**Fig. 1:**
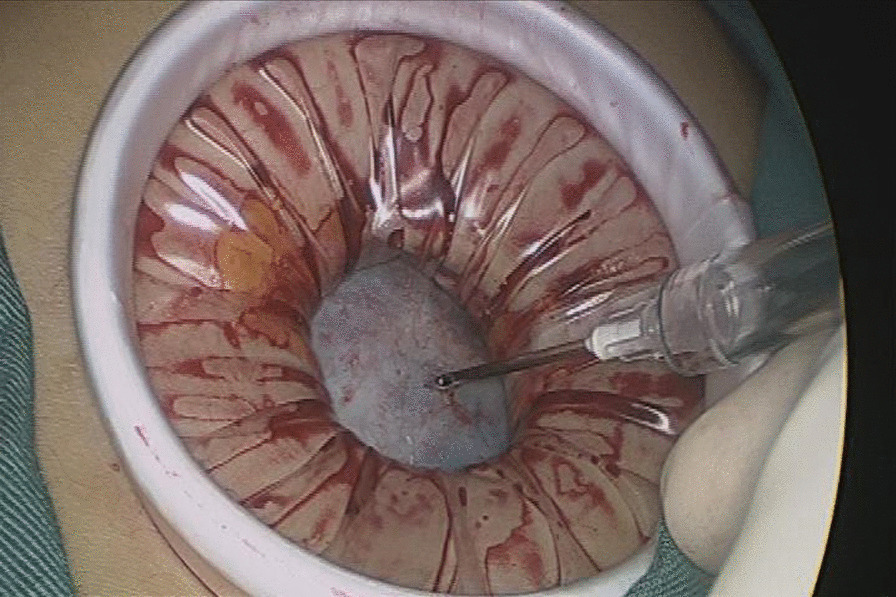
Representative image of aspiration of cyst fluid from giant ovarian cyst

**Fig. 2:**
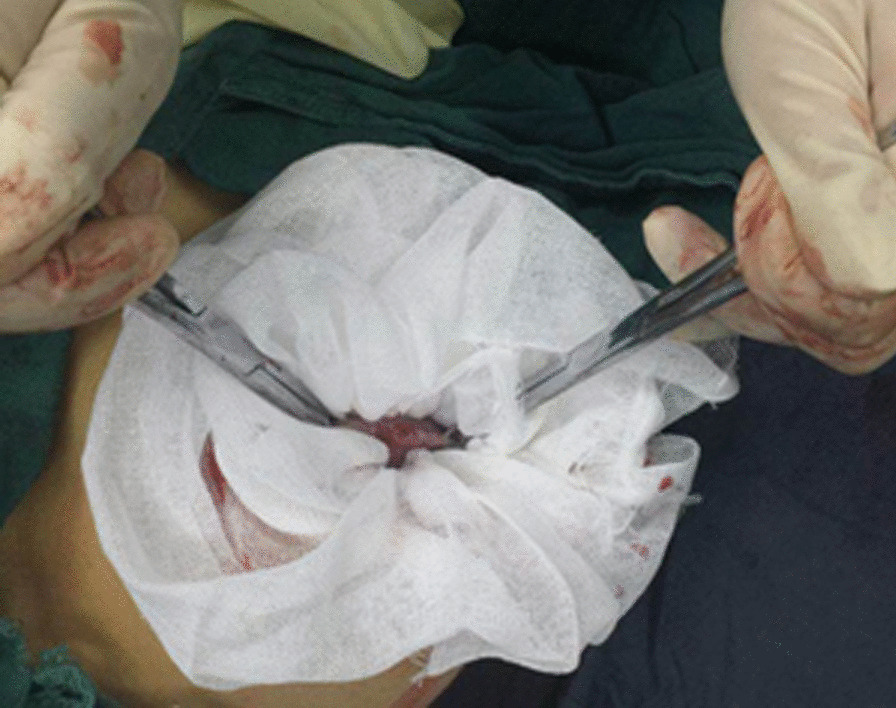
Representative image of decompression of giant ovarian cyst under the protection of surgical gauze

**Fig. 3:**
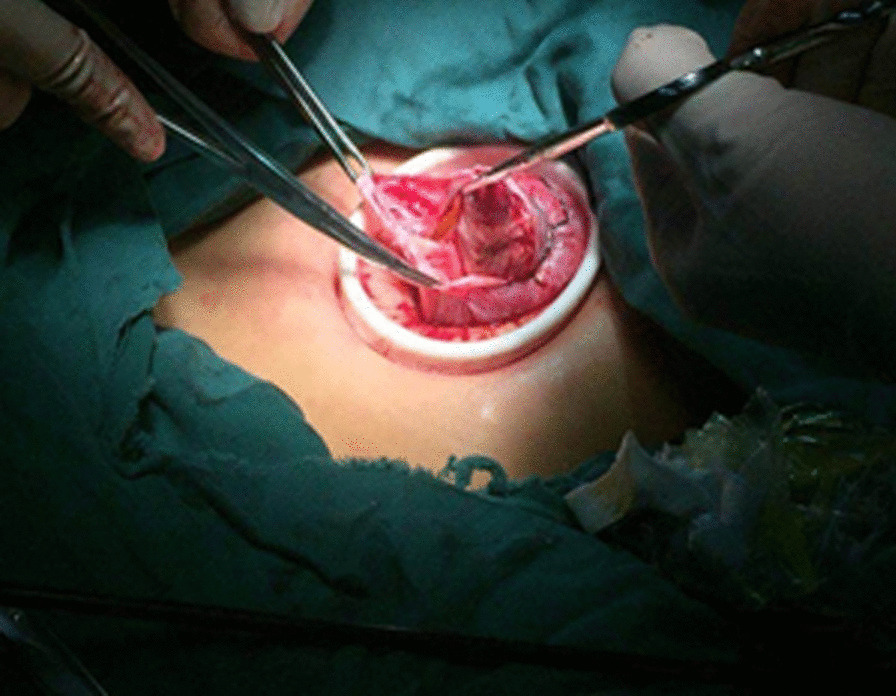
Representative image of the ovarian cyst being pulled out of the extracorporeal space through an umbilical cord incision

**Fig. 4:**
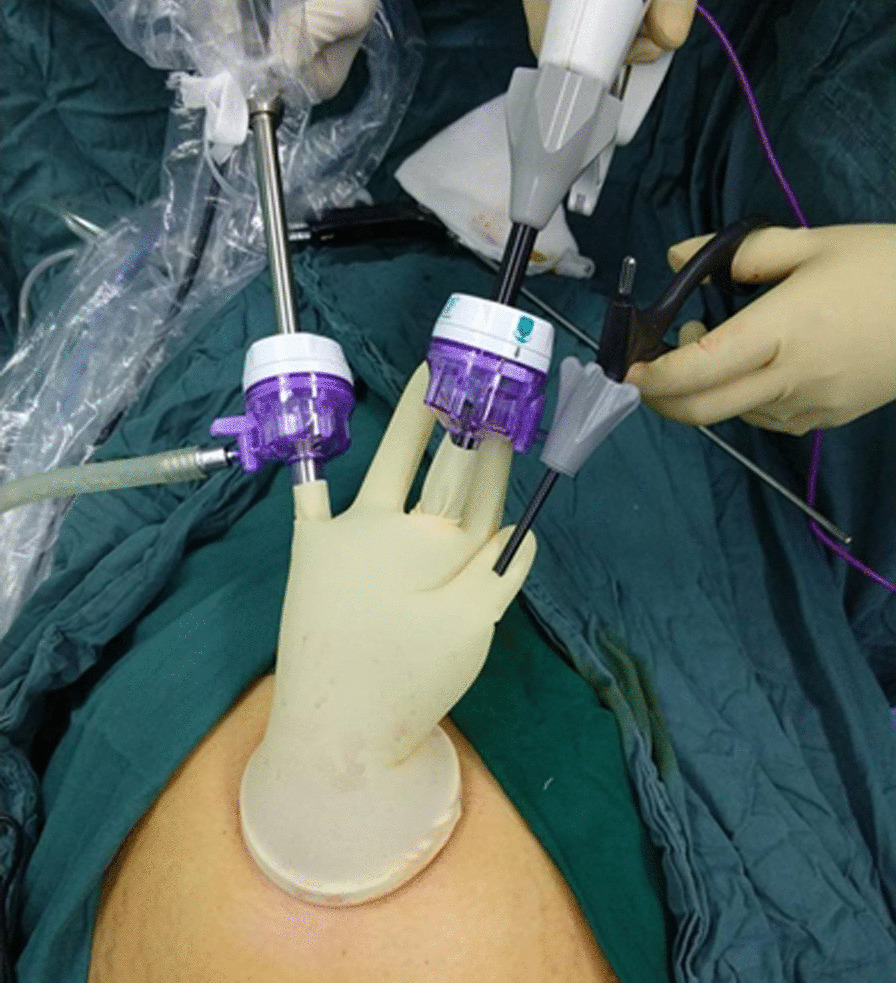
Representative image of the single-port laparoscopic surgery using a home-made multichannel port

**Fig. 5:**
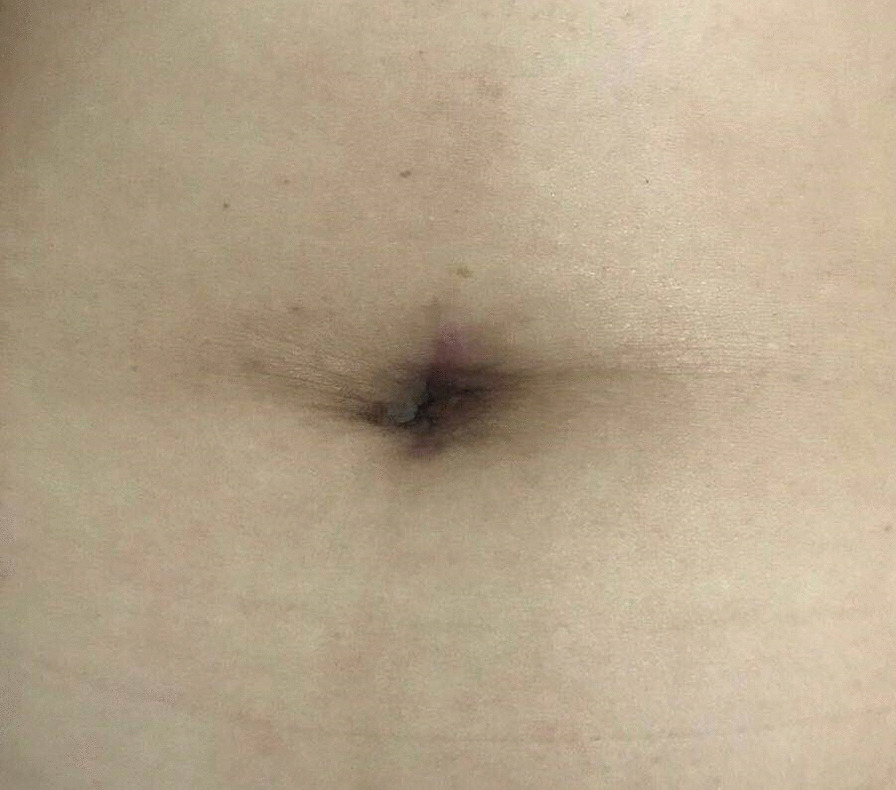
Representative image of the cosmetic effect of umbilical scar after single-port laparoscopic surgery

Patients undergoing single-port laparoscopy received general anesthesia. After cleaning the umbilicus with alcohol swabs and betadine solutions, make an intraumbilical vertical skin incision of about 2 cm, pulled up the umbilicus with the towel clip, and then open the perioneum layer and fascia. The abdomen was insufflated with carbon dioxide gas to maintain intraabdominal pressure at 13 mm Hg. Laparoscope was used to check the free space and the intra-abdominal cavity organs for ascites or tumor metastasis. Remove the laparoscope, aspirate cystic fluid with a suction/irrigation system after puncturing the cyst, and decompress the cyst under the protection of surgical gauze (Figs. 1 and 2). The puncture site was sutured carefully with pulling the cyst capsule continuously, and the ovarian cystic capsule was grasped and pulled upward through the incision site (Fig. 3). The ovarian cyst was carefully removed to avoid spillage. Surgical specimens were sent to the pathology department, and frozen sections were examined for benign or malignant. After completed the ovarian cystectomy, the ovary was reconstituted and returned to the abdominal cavity. The umbilical incision was used to establish the multichannel single-port procedure with a wound retractor and surgical glove (Fig. 4). The finger 1, 3, and 5 were placed with corresponding trocar for laparoscopic instruments. The abdominal cavity was carefully observed for any bleeding lesions, rinsed with 5% warm glucose solution, and then suck out the irrigation fluid in the abdominal cavity. The peritoneum and fascia of the umbilicus and the skin were closed after confirming that there is no bleeding. Cosmetic effect of umbilical cord scar after single-port laparoscopic surgery was shown in Fig. 5.

For three-port laparoscopy, the same preparation maneuver was used. After general anesthesia and making a 1-cm intraumbilical vertical incision, 10 mm trocars were placed. Afterwards, incisions of 10 mm and 5 mm were made in the left and right lower abdomen, and two corresponding trocars were then placed. After the laparoscope and instruments were installed, the ovarian cyst was removed in a process similar to the procedure of single-port laparoscopic surgery. The ovarian cyst was subsequently placed into a laparoscopic retrieval bag to prevent spillage and port-site metastasis. After deflated ovarian cysts were pulled out, the peritoneum and fascia were closed.

For laparotomy, the patient received general anesthesia, followed by a Pfannensteil incision of about 7–8 cm, and then carefully checked the anatomy of the abdomen. The ovarian cyst was removed using an electrosurgical device. The abdominal cavity was carefully examined whether there are any bleeding or pathological lesions.

### Perioperative management and postoperative pain management

Regardless of the surgical methods, postoperative management and postoperative pain management were the same. The perioperative management in each group was in accordance with the ERAS Guideline [10]. Only flurbiprofen (100 mg/day) was used for postoperative pain control, and no other analgesics were used. Pain scores of the patient was evaluated with a Visual analogue scale (VAS) immediately (0 h) after 4 h, 12 h, 24 h, and 48 h after surgery. The higher the score, the more severe the pain.

### Statistical analysis

Statistical analysis was performed using IBM SPSS ver. 19.0 (IBM Co., Armonk, NY, USA). Descriptive data were expressed as mean ± SD. Difference in categorical variables were examined using the Pearson chi-square test. The difference in continuous variables were examined using Student’s *t*-test, one-way ANOVA or Dunnett’s test. A two-tailed *P*-values lower than 0.05 were considered to be statistically significant.

## Results

A total of 95 patients were analyzed in this study, 33 of whom received single-port laparoscopy, 35 patients received laparotomy, and 27 patients received three-port laparoscopy. Table 1 showed the demographics and tumor pathology of patients in the three groups. There was no significant difference in age, BMI, ovarian cyst size, history of abdominal surgery, CA-125 level, ASA classification and pathology of ovarian cyst among the three groups. The average age of patients in the three groups were 31.58, 36.36 and 32.44 years, respectively. The mean BMI of patients in the three groups were 25.09, 23.9 and 23.6 kg/m^2^, respectively. The mean diameter of the ovarian cyst in the three groups were 17.36, 18.11 and 16.33 cm, respectively. There was no significant difference between the three groups in the CA-125 levels (40.89, 51.76 and 27.84, respectively) and ASA Classification (1.79, 1.91 and 1.74, respectively). Pathology of ovarian cysts in the single-port laparoscopy group included 9 serous cystadenoma (27%), 11 mucinous cystadenoma (33%), 8 ovarian teratoma (24%), 3 endometriotic cyst (9%), and 2 fibrothecoma (6%). Laparotomy group included 12 serous cystadenoma (34%), 16 mucinous cystadenoma (46%), 4 ovarian teratoma (11%), and 3 endometriotic cyst (9%). Three-port laparoscopy group included 8 serous cystadenoma (30%), 11 mucinous cystadenoma (41%), 3 ovarian teratoma (11%), and 5 endometriotic cyst (19%).

**Table 1:** Demographics and tumor pathology of patients for single-port laparoscopy, laparotomy and three-port laparoscopy (n = 95)

Characteristics	Single-port Laparoscopy	Laparotomy	Three-portLaparoscopy	*P*-value
Age (year)	31.58 ± 11.73	36.34 ± 18.09	32.44 ± 12.53	0.06
BMI (kg/m^2^)	25.09 ± 4.56	23.9 ± 4.70	23.6 ± 4.25	0.71
Previous abdominal surgery	0.15 ± 0.36	0.26 ± 0.56	0.29 ± 0.47	0.46
Ovarian cyst diameter (cm)	17.36 ± 4.07	18.11 ± 4.11	16.33 ± 2.09	0.06
CA-125 (U/ml)	40.89 ± 97.76	51.76 ± 74.37	27.84 ± 18.44	0.80
ASA Classification	1.79 ± 0.42	1.91 ± 0.45	1.74 ± 0.45	0.26
Pathology of ovarian cyst				
Serouscystadenoma	9 (27%)	12 (34%)	8 (30%)	
Mucinouscystadenoma	11 (33%)	16 (46%)	11 (41%)	
Ovarianteratoma	8 (24%)	4 (11%)	3 (11%)	
Endometrioticcyst	3 (9%)	3 (9%)	5 (19%)	
Fibrothecoma	2 (6%)	0 (0%)	0 (0%)	

*BMI* Body mass index, *ASA* *Classification* ASA Physical Status Classification, *CA-125* Cancer antigen 125

Values were presented as mean ± SD or number (%)

The perioperative surgical outcomes and complications were shown in Table 2. The mean operation time, estimated blood loss, hemoglobin changes, and tumor spillage in the three-port laparoscopy group were significantly higher than that in the single-port laparoscopy. The estimated blood loss in the single-port and three-port laparoscopy were 28.91 ml and 63.7 ml, respectively. The hemoglobin changes in the single-port and three-port laparoscopy were 18.58 and 26.93 g/dL, respectively. Six patients (22.2%) in the three-port laparoscopy group experienced tumor rupture with spillage, while only 1 patient (3.0%) in the single-port laparoscopy group and 1 patient (2.9%) in the laparotomy group had tumor spillage during surgery. This seems to indicate that three-port laparoscopy has a higher risk of tumor rupture than single-port laparoscopy and laparotomy in removing giant ovarian cysts larger than 15 cm. On the other hand, all postoperative variants between groups were analyzed and showed that single-port laparoscopy had shorter recovery time than three-port laparoscopy and laparotomy. The time until to gas passing in the single-port laparoscopy was significant shorter than that in the laparotomy (41.53 vs 47.31 h, *P* < 0.05). In addition, single-port laparoscopy showed the shortest time interval to leave bed (the first time out of bed for activity) than three-port laparoscopy (17.53 vs 24.56 h, *P* < 0.01) and laparotomy (17.53 vs 29.40 h, *P* < 0.01). As expected, the length of hospital stay in the single-port laparoscopy was significantly shorter than that in the laparotomy (4.06 vs 5.46 days, *P* < 0.001) and three-port laparoscopy (4.06 vs 4.81 days, P < 0.001). Although single-port laparoscopy has better postoperative outcomes, there were no significant differences in the total hospital cost between the three groups. On the other hand, although there was no significant difference in postoperative complications between the three groups (P > 0.05), laparotomy did show a higher risk of postoperative complications than single-port laparoscopy and three-port laparoscopy (14.3% vs 6% vs 6%). After single-port or three-port laparoscopy, only a few patients developed fever, ileus and intra-abdominal bleeding. In addition, during single-port laparoscopic or three-port laparoscopic surgery, no patient experienced additional port insertion or conversion to laparotomy.

**Table 2:** Comparison of perioperative surgical outcomes and complications (n = 95)

Variants	Single-port Laparoscopy	Laparotomy	Three-port Laparoscopy
Intraoperative			
Operation time (min)	73.91 ± 20.54*	66.57 ± 40.43	88.33 ± 33.69
Estimated blood loss (ml)	28.91 ± 23.61*	29.71 ± 25.35	63.7 ± 94.01
Hemoglobin changes (g/dL)	18.58 ± 12.83**	15.80 ± 8.65	26.93 ± 10.45
Cyst rupture during operation	1 (3.0%)*	1 (2.9%)	6 (22.2%)
Postoperative			
Time until gas passing (hour)	41.53 ± 8.27^†^	47.31 ± 13.33	41.33 ± 10.08
Time until leaving bed (hour)	17.53 ± 7.26^††,^**	29.40 ± 9.57	24.56 ± 7.76
Length of hospital stay (day)	4.06 ± 0.50^†††,^***	5.46 ± 1.63	4.81 ± 0.83
Total hospital cost (US dollar)	3554.3 ± 447.9	3392.3 ± 1224.9	3889.5 ± 970.6
Complications	2 (6.0%)	5 (14.3%)	2 (7.4%)
Fever	1 (3.0%)	3 (8.6%)	2 (7.4%)
Ileus	0 (0.0%)	1 (2.9%)	0 (0.0%)
Intra-abdominal bleeding	1 (3.0%)	1 (2.9%)	0 (0.0%)

Values were presented as mean ± SD and number (%)

*Denoted a statistically significant difference between single-port and three-port laparoscopy

^†^Denoted a statistically significant difference between single-port laparoscopy and laparotomy

*, **, and ***Denoted *P* < 0.05, *P* < 0.01, and *P* < 0.001, respectively

^†^, ††, and †††Denoted *P* < 0.05, *P* < 0.01, and *P* < 0.001, respectively

During the recovery period, there was significant postoperative pain among the three groups (Table 3). Compared with laparotomy and three-port laparoscopy, patients underwent single-port laparoscopy had the lowest postoperative pain scores at 0, 4, 8, 12 and 24 h. After 24 and 48 h after the surgery, there was no significant difference in postoperative pain between single-port laparoscopy and three-port laparoscopy.

**Table 3:** Postoperative pain score

	Single-port Laparoscopy	Laparotomy	Three-port Laparoscopy
Pain scores during recovery			
Immediately after operation	3.12 ± 0.86^†††^,*	3.83 ± 0.57	3.52 ± 0.51
4 h postoperation	2.70 ± 0.85^†††,^**	3.37 ± 0.65	3.07 ± 0.38
8 h postoperation	2.70 ± 0.85^†††,^**	3.37 ± 0.65	3.07 ± 0.38
24 h postoperation	1.94 ± 0.61^†††^	2.63 ± 0.65	1.81 ± 0.48
48 h postoperation	1.36 ± 0.55^†††^	2.13 ± 0.49	1.51 ± 0.58

Pain scores were evaluated using VAS after surgery. Values were presented as mean ± SD

*Denoted a significant statistical difference between single-port and three-port laparoscopy groups. * and **Denoted *P* < 0.05 and *P* < 0.01, respectively. ^†^Represented a significant statistical difference between single-port laparoscopy and laparotomy groups. ^†††^ denotes *P* < 0.001

## Discussion

Today, laparoscopy has become one of the standards for removing ovarian tumors, because laparoscopy has obvious advantages in cosmetic, perioperative and postoperative outcomes and complications. With the development and advances in surgical instruments and technology, surgeons have attempted to reduce the port numbers and to treat larger ovarian tumors. Although reducing port numbers can achieve better cosmetic outcomes, it is a highly challenging technology. Reducing the number of ports also means that the instruments are crowded around the surgical site; the number of available instruments during surgery are limited; a longer operating time is required; and technical learning of clinicians will be relatively long [9, 11]. Another concern of reduced port surgery is the tumor rupture and spillage, which may increase the risk of tumor progression. The results of this comparative study further showed that single-port laparoscopy is safe and feasible for ovarian cysts larger than 15 cm, with shorter operation time, less estimated blood loss, lower hemoglobin changes, and smaller tumor spillage report. In addition to better cosmetic effects, single-port laparoscopy has shorter the hospital stay and postoperative pain than three-port laparoscopy and laparostomy.

For larger ovarian tumors, single-port laparoscopic surgery is more concerned about the increased risks of tumor rupture and spillage of ovarian cyst fluid, such as the spread of malignant tumor cells, recurrence, pseudomyxoma peritonei, peritonitis, and gliomatosis peritonei [12, 13]. It was reported that the rate of ovarian tumor rupture during laparoscopic surgery is about 6–27% [11, 14–17] and even more than 60% [9]. However, several studies indicated that intraoperative tumor rupture may not increase the incidence of relapse or prognosis. Study by Dembo et al. demonstrated that tumor grade, dense adhesions and ascites, rather than tumor spillage, were crucial factors affecting tumor relapse [18]. Intraoperative tumor rupture did not associated with the survival rate [19, 20], and recurrence rate [17]. However, these patients with intraoperative tumor rupture received further postoperative adjuvant radiotherapy [19, 20], or neoadjuvant BEP (cisplatin, etoposide, and bleomycin) treatment [17]. On the other hand, other studies have reported different findings. Intraoperative tumor rupture may worsen the prognosis and reduce overall survival (OS), disease-free survival (DFS) or cancer-specific survival (CSS) [21–24]. The results of this study showed that a lowest incidence rate of 3.0% (1/33) was observed in the single-port laparoscopic surgery group, which was much lower than the 22.2% (6/27) in the three-port laparoscopic surgery group. Similar observation was observed in ovarian tumors < 15 cm by Roh et al.[8] and Chong et al.[7] This study further indicated that even for giant ovarian tumors (> 15 cm), single-port laparoscopy still has obvious advantages over three-port laparoscopy and laparostomy in reducing the spillage rate of tumor fluid.

A retrospective study of 186 cases of ovarian cyst removal by Hizkiyahu et al. showed that larger ovarian cysts are positively associated with the occurrence of intraoperative cyst spillage [25]. In addition, the use of endoscopic retrieval bag is negatively associated with the risk of cyst spillage. Another study of 53 young patients by Yousef et al. also supported that rupture of pediatric ovarian neoplasms was associated with increasing cyst size [17]. On the other hand, spillage of ovarian cyst content may increase the risk of spread of malignant cells and tumor recurrence. Ben-Ami et al. retrospectively analyzed the clinical and surgical characteristics of 42 women who underwent surgical removal of benign cyst, and found that intraoperative cyst rupture was significantly associated with cyst recurrence [26]. Due to the recurrence of the cyst, three women underwent a second operation. It is worth noting that these three women with recurrent cysts ruptured during the operation. A recent meta-analysis [27] also concluded that intraoperative cyst spillage is associated with increased risk for benign recurrence (RR 3.1; 95% CI, 1.05–9.14). Thus, no matter which surgical approach is used, maximal efforts should be made to prevent intraoperative cyst rupture and spillage. In the single-port laparoscopy group of this study, patients with cyst ruptures were constantly aspirating near the site of the puncture site and undergoing copious peritoneal lavage and drainage to minimize the risk of spillage. For patients in the three-port laparoscopy group, laparoscopic retrieval bag was used to prevent spillage and port-site metastasis. The abdominal cavity was irrigated when the ovarian cyst spillage during surgery.

The results of our study showed that the operation time and estimated blood loss of the single-port laparoscopy were significantly shorter than that of the three-port laparoscopy. However, other studies reported that there were no significant differences in the perioperative outcomes, such as operating time, estimated blood loss and changes of hemoglobin level [28–30]. Kim et al. in Korea compared single-port, two-port and four-port laparoscopic surgery for cyst enucleation in benign ovarian cysts with size 6.3–7.5 cm [6]. In contrast, single-port laparoscopic surgery had a significant longer operation time and a higher estimated blood loss than two-port and four-port laparoscopic surgery. Different to our single-port laparoscopy, Kim’s team used a homemade single-port device with a wound retractor. They demonstrated that single-port surgery was performed by inserting instruments through the umbilicus incision, making it difficult to perform operations including ligation or suture. However, study by Yim et al. in Korea [31] showed that there was no significant difference in the operation time between single-port laparoscopy and laparoscopy for 5–9 cm tumor sizes. But the estimated blood loss in single-port laparoscopy was significantly lower than that in laparoscopy. Recently, the same team used single-port laparoscopy with SW Kim’s technique for huge ovarian tumors (17 cm), of which were placed in a laparoscopic bag (LapBag) [9]. Single-port laparoscopy still has lower estimated blood loss than that in laparostomy. Even though there was no statistical significance, single-port laparoscopy did have a short operation time than laparostomy (86.0 vs 107.5, P = 0.142). It is likely that the increased in the ovarian tumor size may prolong the operation time and increase the estimated blood loss in the three-port laparoscopy, but not in single-port laparoscopy. The difference in single-port laparoscopy techniques may also be one of the possible explanations. In this study, the giant ovarian tumors were pulled out through the umbilical incision for cyst removal and suture, and the process of hemostasis was relative clear and easy. For three-port laparoscopy or single-port laparoscopy without aspiration, due to the giant ovarian tumors, the free-space in the abdominal cavity is relatively reduced, which further increases the interference of surgical instruments within the abdominal cavity. Therefore, it may take a relatively long time to remove the cyst through the abdominal trocars.

Another finding of this study was that single-port laparoscopy significantly reduced postoperative pain and time interval to leave the bed, and shorten the hospital stay, which is supported by other studies [9, 32]. Kim et al. showed that immediate postoperative pain scores of patients in the single-port laparoscopy group was lower than that in the laparotomy group. However, there were no statistical difference in postoperative pain scores between single-port laparoscopy and laparotomy 6 h after surgery. It is worth noting that even 48 h after surgery, the postoperative pain scores of our laparoscopic surgery was still significantly lower than that of laparotomy. Compared with the three-port laparoscopy, our single-port laparoscopic surgery has a significant lower pain score at 0, 4 and 8 h after surgery. These outcomes may also direct/indirectly contribute to shorten the on-bed time of patients and the length of hospital stay. Studies have shown that leaving bed for activities early can increase lung capacity, help lung expansion and reduce lung complications, promote the metabolism of the body and recovery of intestinal peristalsis, reduce intestinal adhesion, and avoid the formation of deep vein thrombosis, thereby reducing the occurrence postoperative complication [33, 34]. Although there was no statistical difference, the single-port laparoscopic surgery in this study had the lowest postoperative complication rate. Future large-scale population trials are warranted to better explore the benefits of single-port laparoscopic surgery in terms of postoperative complications and other outcomes.

The present study has several limitations. Since giant ovarian cysts larger than 15 cm are not common, one of the limitations of this study is the small sample size. Another limitation is due to its retrospective design, we cannot exclude its selection bias and different physician experience. Although we attempted to control for case complexity, the experience of surgeon is difficult to measure. In order to improve the above limitations of this study, randomized prospective studies with a large number of patients are needed in the future.

## Conclusions

Compared with three-port laparoscopy and laparostomy, single-port laparoscopy can significantly reduce the operation time, estimated blood loss and tumor spillage for giant ovarian tumors larger than 15 cm. In addition, single-port laparoscopy has the benefits of less postoperative pain and shorter length of hospital stay. It is important that single-port laparoscopy will not increase the patient’s total hospital costs and postoperative complications. Thus, single-port laparoscopy is a safe and efficient technique for removal of a giant ovarian tumors. Future multicenter randomized controlled trials are warranted to further prove the benefits and safety of single-port laparoscopy.

## Data Availability

The data used and analyzed in this study are available from the corresponding author upon reasonable request.

## Abbreviations

BMI: Body mass index
ERAS: Enhanced Recovery After Surgery
VAS: Visual analogue scale
OS: Overall survival
DFS: Disease-free survival
CSS: Cancer-specific survival
LapBag: Laparoscopic bag
